# Isolated spinal aneurysms with spontaneous regression

**DOI:** 10.1007/s10143-025-03768-8

**Published:** 2025-09-08

**Authors:** Vivien Richter, Ali Khanafer, Sven Poli, Alexandra Gómez Expósito, Patrick Haas, Constantin Roder, Ulrike Ernemann, Hans Henkes, Florian Hennersdorf

**Affiliations:** 1 Department of Diagnostic and Interventional Neuroradiology, University Hospital Tübingen, Tübingen, Germany; 2 Neuroradiology Clinic, Klinikum Stuttgart, Stuttgart, Germany; 3 Department of Neurology & Stroke, Hertie Institute for Clinical Brain Research, Eberhard-Karls-University Tübingen, Tübingen, Germany; 4 Department of Neurosurgery and Neurotechnology, University Hospital Tübingen, Eberhard Karls University Tübingen, Hoppe-Seyler Street 3, Tübingen, Germany

**Keywords:** Spinal aneurysm, Spinal hemorrhage, Subarachnoid hemorrhage, Spinal angiography

## Abstract

**Purpose:**

To share our clinical experience with conservative management of isolated spinal arterial aneurysms (ISAs) and to identify clinical scenarios where conservative management may be appropriate, in the context of a literature review.

**Methods:**

We performed a retrospective review of spinal angiograms from two German neuroradiology centers and conducted a systematic literature review of reported ISA cases. We analyzed demographics, clinical presentation, imaging findings, treatments, and outcomes.

**Results:**

We identified seven patients (mean age 48; 4 women) with nine ISAs, eight of which were managed conservatively. Five of them had excellent short-term outcomes, and spontaneous regression was documented in four cases. With our cases, 208 ISAs have been reported in the literature in 164 patients (mean age 51; 52% female). They most commonly present with subarachnoid hemorrhage (90%) and back pain (69%). Most are located in the thoracic (51%) or cervical spine (40%) and involve the anterior spinal circulation (63%). They may be treated by surgical intervention (47%) or conservatively (37%), less commonly by endovascular therapy (16%). 78% of patients have favorable outcomes (mRS 0–3), similar across all treatment approaches. In 52% of conservatively managed cases, spontaneous regression was documented by imaging. Clinical deterioration was primarily associated with respiratory complications and spinal cord infarction, with a documented rebleeding rate of 8% and a mortality rate of 12%.

**Conclusions:**

ISAs are a rare and potentially underrecognized cause of cerebral and spinal subarachnoid hemorrhage. In select cases, particularly small ISAs of the anterior spinal or a radiculomedullary artery and with transient neurological symptoms, conservative management appears to be a reasonable approach, supported by increasing evidence of the possibility of spontaneous regression.

**Supplementary Information:**

The online version contains supplementary material available at 10.1007/s10143-025-03768-8.

## Introduction

Isolated spinal aneurysms (ISAs) are a rare and often underrecognized cause of cerebral or spinal subarachnoid hemorrhage (SAH) [1]. In cases of infratentorial cerebral SAH, the spinal vasculature may be overlooked as a possible source of bleeding. ISAs may be underreported due to rarity and subtle imaging findings seen only on MRI and spinal angiograms.

The causes of a spinal SAH can be broadly classified into five categories: spontaneous hemorrhage (e.g., coagulopathies, anticoagulation overdosage); trauma; iatrogenic causes; vascular anomalies; and neoplasms [2]. Vascular anomalies include arterio-venous malformations (AVMs); arteriovenous fistulas (AVFs); neoplastic lesions (e.g., hemangioblastomas and cavernous malformations), and ISAs [3]. 

ISAs can arise from any major spinal artery, including the anterior spinal artery (ASA), posterior spinal arteries (PSAs), radiculomedullary arteries (RMAs), radicular arteries, or the artery of Adamkiewicz.

Predisposing factors include aortic coarctation, drug abuse, infections, previous trauma, severe vomiting, pregnancy, and connective tissue disorders [4]. 

Management of ISAs must be individualized, considering the patient´s clinical status, the severity and complications of the hemorrhage, aneurysm location and configuration, flow dynamics and downflow pattern of the parent artery, and the expertise available at the treating center.

## Materials and methods

A retrospective review of all spinal angiograms at two high-volume neuroradiology institutes was performed to identify cases with ISAs. The Institutional Review Board approved the retrospective study with a waiver for individual patient consent. The systematic literature review was conducted per the Preferred Reporting Items for Systematic Reviews and Meta-Analyses (PRISMA) guideline [5]. A comprehensive search was performed in the PubMed, Ovid MEDLINE, and Google Scholar databases from their inception through 1 June 2024. Search terms and their derived MeSH and keywords included a combination of “spinal aneurysm”, “spinal artery aneurysm”, “radicular artery aneurysm”, “radiculomedullary artery aneurysm”, “lateral spinal artery aneurysm”, “posterior spinal artery aneurysm”, “anterior spinal artery aneurysm”, and “artery of Adamkiewicz aneurysm”. Search results were imported and merged into a reference management software (Endnote v20.6, Clarivate Analytics, Philadelphia, PA, USA). Reference lists of the included articles were also reviewed. Duplicates were removed, the remaining titles and abstracts were assessed, and articles irrelevant to the topic, not in English or German, or not retrievable were excluded. Full texts of relevant articles were retrieved and reviewed. Data collected included patient age, gender, comorbidities, presenting symptoms, aneurysm location, configuration, multiplicity, treatment approaches, clinical and imaging follow-up, and outcomes. All collected data was analyzed in JMP 16.2.0 (SAS Institute, USA).

## Results

### Case series

**Fig. 1:**
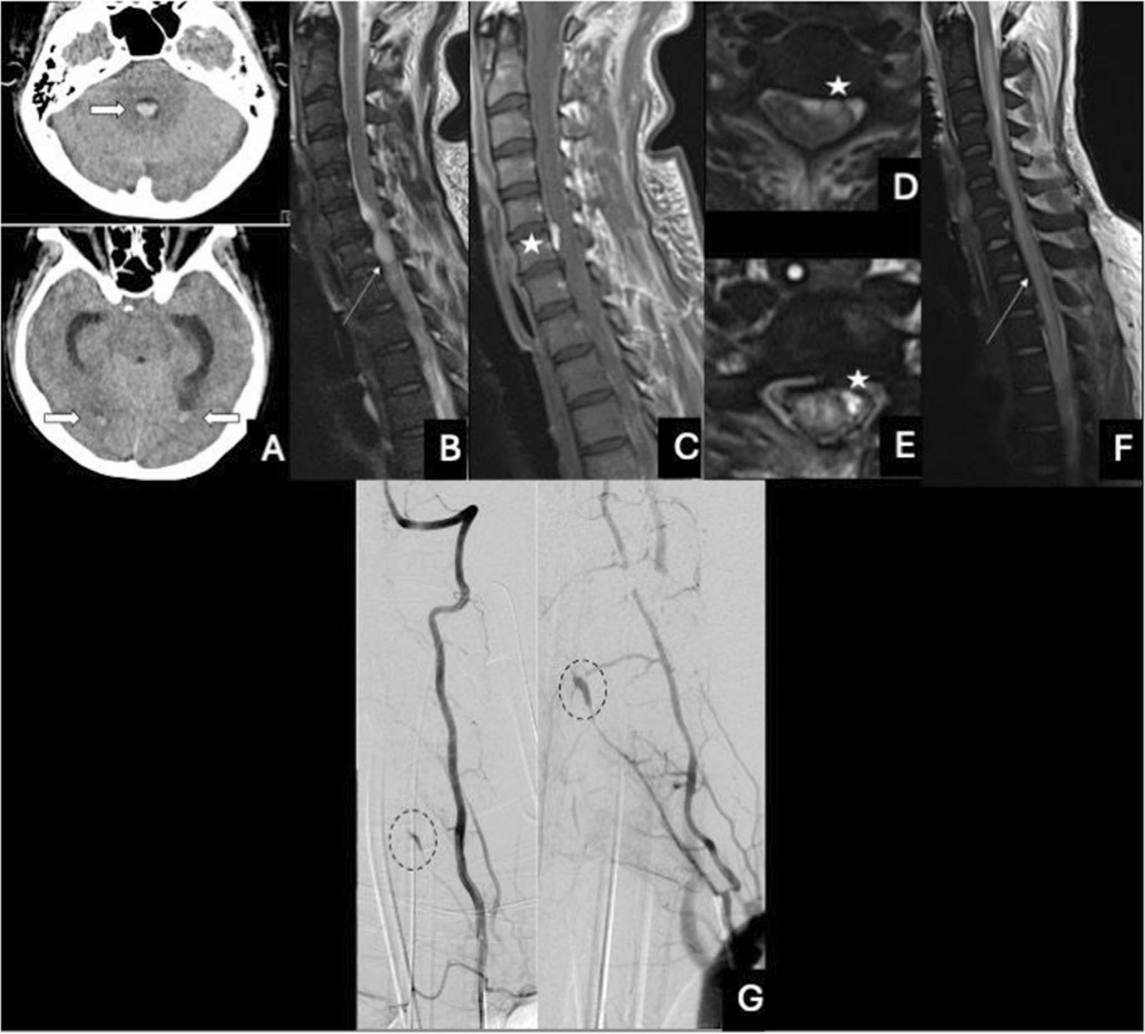
Representative case (Patient 1). A) Head CT showed minimal basal SAH. B-F) Spinal MRI showed extensive cervical and intracranial SAH as seen on sagittal T2 (B) and axial T2 (D) weighted images, as well as an intradural cystic mass ventral to the cervical myelon as seen on sagittal (C) and axial (E) contrast-T1 weighted images. Extensive spinal cord infarction is seen on sagittal T2 (F). A fusiform aneurysm of a cervical RMA at C7 level was found on DSA, with supply from the left inferior thyroid artery and the cervical ascendent artery (G)

**Fig. 2:**
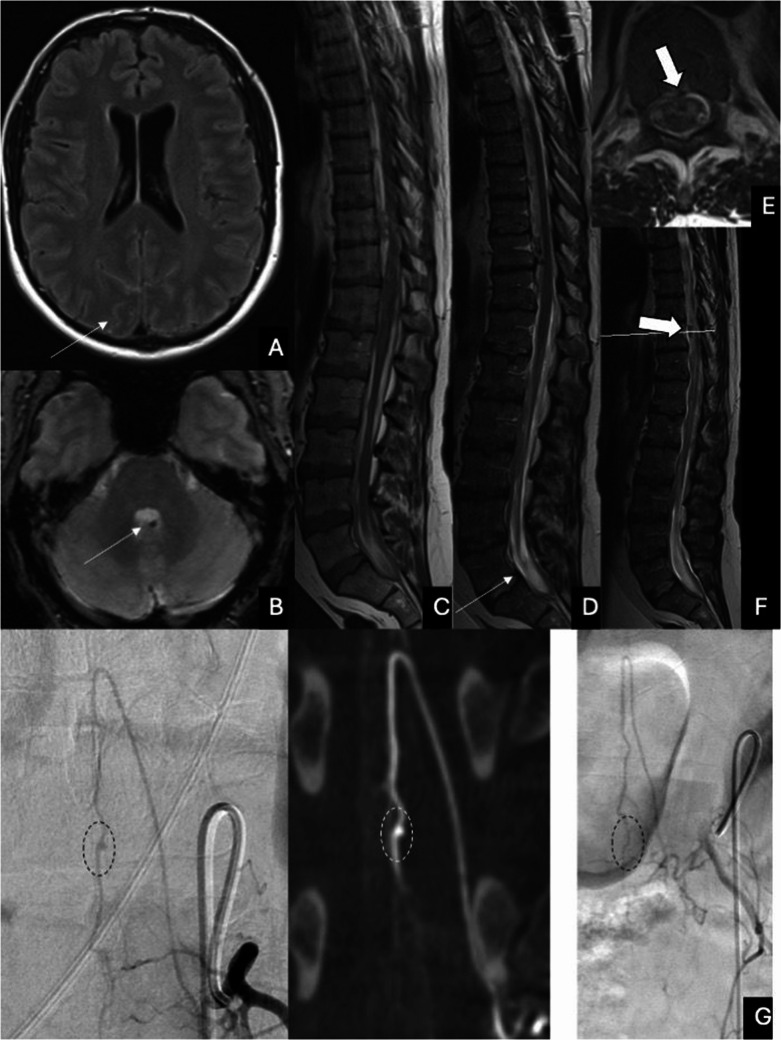
Representative case (Patient 4). On cranial MRI, cerebral SAH is seen on FLAIR (A) and T2* (B) images. On spinal MRI, spinal SAH is seen in the thecal sac (C and D), and a small cystic lesion with flow void in the ventral dural sac at T11 level (E and F). DSA shows a fusiform aneurysm of the ASA at T12 level (G)

We identified seven patients with a total of nine ISAs in a retrospective database review. Detailed patient data is provided in Table 1, and representative imaging is shown in Figs. 1 and 2.

**Table 1:** Patient characteristics, imaging findings, treatment approach, and outcome. (C: cranial CT, sMRI: spinal MRI, cMRI: cranial MRI, DSA: digital Subtraction angiography; RMA: radicular-medullary artery, ASA: anterior spinal artery, PSA: posterior spinal artery, VA: vertebral artery; SAH: subarachnoid hemorrhage, EDH: epidural hemorrhage. SDH: subdural hemorrhage; mRS: modified Rankin score.)

Patient	Age (years)	Gender	Relevant comorbidities	Presenting symptoms	Imaging findings	ISA level	Treatment	Outcome
**1**	41	female	Systemic lupus erythematosus with glomerulonephritis and anticoagulation for a previous pulmonary embolism.	First episode of grand mal seizure, loss of consciousness, subsequent lower extremity paraplegia.	cCT: unremarkable. sMRI: extensive cervical and intracranial SAH; intradural cystic mass ventral to the cervical myelon, extensive spinal cord infarction. DSA: fusiform aneurysm of an RMA, with supply from the left inferior thyroid artery and the cervical ascendent artery	C7	Best medical treatment Surgical decompression of hemorrhage	mRS = 5 at discharge, no follow-up available
**2**	34	male	Cocaine abuse	Acute thunderclap-like episode of back pain in the thoracic region, neck stiffness	cCT: unremarkable. sMRI: minimal SAH in the thecal sac; tubular contrast-enhancing structure at C5 level within the ventral dural sac corresponding to a ruptured aneurysm. DSA: fusiform aneurysm an RMA originating from the right VA and supply of the ASA.	C5	Best medical treatment	mRS = 0 at discharge, no follow-up available
**3**	38	female	none	Acute thunderclap-like episode of back pain in the thoracic region, neck stiffness.	cCT: unremarkable. sMRI: extensive spinal SAH and a mid-thoracic 2 mm nodular contrast-enhancing lesion. DSA: 2 fusiform aneurysms of an RMA supplying the ASA.	T5	Best medical treatment	mRS = 0 at discharge and at 3 months follow-up
**4**	46	male	none	Acute thunderclap-like episode of back pain with an ascending character, transient lower extremity paraparesis.	sMRI: cerebral and spinal SAH, small cystic lesion with flow void in the ventral dural sac at T11 level. DSA: 1 fusiform aneurysm of the ASA.	T12	Best medical treatment	mRS = 0 at discharge and at 3 months follow-up
**5**	84	Female	oral anticoagulation for atrial fibrillation	Acute thunderclap-like episode of back pain, lower extremity paraparesis, urinary incontinence.	cMRI: unremarkable. sMRI: extensive spinal SAH, extensive spinal infarction, cystic contrast-enhancing lesion in the ventral dural sac at T1 level. DSA: 1 fusiform aneurysm of an RMA supplying the ASA l, with partial thrombosis.	T2	Best medical treatment	mRS = 4 at discharge, no follow-up available
**6**	40	male	multiple drug abuse (drugs not identifiable)	Acute thunderclap-like episode of back pain, lower extremity paraparesis, hypoesthesia.	sMRI: lumbar spinal SAH and EDH, nodular lesion at the L1 level. DSA: 1 small fusiform aneurysm of a PSA at L1 level, unchanged on follow-up DSA at day 14.	L1	Best medical treatment.	mRS = 0 at discharge and at 5 months follow-up
**7**	51	female	Cerebral AVM	Acute thunderclap-like episode of back pain, urinary incontinence, bowel dysfunction, upper and lower extremity hypoesthesia	sMRI: spinal SAH and SDH. DSA: 1 fusiform aneurysm of a PSA at T5 level, unchanged on follow-up DSA at day 14. 1 fusiform aneurysm of an RMA supplying the ASA at T7 level, undetectable on follow-up DSA at day 14.	T5 T7	Best medical treatment (RMA) Surgical wrapping (PSA)	mRS = 0 at discharge and at 3 months follow-up

All aneurysms presented with a symptomatic SAH, symptoms being headaches, neck stiffness, thunderclap-like back pain, or focal neurological deficits.

The parent artery was an RMA in 6 out of 9 ISAs, with some of these RMAs supplying the ASA. The remaining ISAs originated from a PSA in 2 cases and the ASA itself in 1 case. 7 out of 9 ISAs were in a thoracolumbar location.

Most aneurysms (7 out of 9) were managed conservatively with the best medical treatment. Surgical decompression and local coagulation were performed in one case with severe hemorrhage and neurological impairment; the small ISA was not identifiable on surgery. In one case, a PSA aneurysm was still identifiable on follow-up DSA and was subsequently treated surgically. Spontaneous regression was documented in 4 aneurysms.

A good short-term functional outcome (mRS 0–3) was reported in five out of seven patients. Follow-up at 3 to 5 months was available in four of our patients, all of whom showed no clinical change and an excellent outcome.

### Results of the literature review

127 relevant publications were published between 1903 and 2024, comprising 157 patients with a total of 197 ISAs. Notably, 75% of these publications appeared after 2005, reflecting increased recognition and reporting in recent years. The workflow of the systematic literature review following the PRISMA guideline can be found in Suppl. 1. A summary of the aggregated data is presented in Table 2; not all data points were consistently reported across all included studies. The full list of references is provided in Suppl. 2.

**Table 2:** Results of the literature review

Review			
Number of aneurysms			*N* = 208
Number of patients			*N* = 164
Number of publications			*N* = 127
Mean age			51 years
Gender			52% female
			48% male
Ruptured with SAH			90%
Patients with multiple ISAs			9%
Location		Cervical	40%
		Thoracic	51%
		Lumbosacral	9%
Parent artery		Anterior spinal artery	31%
		Posterior spinal artery	13%
		Lateral spinal artery	5%
		Artery of Adamkiewicz	5%
		Radiculomedullary artery	32%
		Other	14%
Treatment of aneurysm		Surgical	47%
		Endovascular	16%
		Best medical treatment	37%
Clinical outcome	all	Good/fair	79%
		Poor	9%
		Death	12%
	surgery	Good/fair	87%
		Poor	8%
		Death	5%
	endovascular	Good/fair	75%
		Poor	18%
		Death	7%
	conservative or no treatment prior to death	Good/fair	69%
		Poor	6%
		Death	24%
Spontaneous regression documented		conservative or no treatment due to death	52%

### Baseline characteristics and etiology

There was considerable inconsistency in the reporting of the parent artery in the literature. The most common parent artery was the ASA or an RMA, each accounting for approximately 30% of cases, followed by a PSA in 13%. Most aneurysms (82%) were fusiform.

Among unruptured ISAs, symptoms were primarily due to mass effect (11 cases) or spinal cord ischemia (2 cases). In ruptured cases, subdural hematoma (2 cases), epidural hematoma (1 case), and intramedullary hematoma (1 case) was observed rarely besides a SAH.

The most frequently reported clinical symptoms at presentation were acute thunderclap-like back pain (69%), focal neurological deficits (68%), headache (46%) and vomiting (25%). Cervical ISAs were more commonly associated with infratentorial SAH, headache, nausea, and vomiting, whereas thoracic ISAs were more likely to present with acute back pain. The prevalence of focal neurological deficits was similar across all spinal levels.

42% of the patients were previously healthy with no identified risk factors. Hypertension was the most frequently reported potential contributor (15 cases). Other associated conditions included aortic coarctation (13 cases), vasculitis (8 cases), bilateral vertebral artery occlusion with flow diversion to the ASA (7 cases) and altered vascular anatomy following organ transplantation (7 renal and 3 cardiac transplant recipients). Drug abuse was reported in 6 cases. Five cases occurred in the postpartum period, and one during pregnancy. In some instances, aneurysm rupture was preceded by activities that likely increased spinal pressure: severe vomiting and retching in four cases and severe coughing in one case.

### Management and outcomes

37% of the published cases were managed conservatively, and 63% were treated by surgical (72%) or endovascular (38%) intervention. Coiling was used in 21 cases (65%), glue embolization in six (19%), particles in three, and liquid embolization with Onyx in two cases. There was no statistical correlation between demographic, clinical, or morphologic factors and choice of treatment approach.

Clinical outcome was good/fair (mRS 0–3) in 78%. Rebleed was observed in 8% of all cases (14 cases) and in 7% in the conservatively managed subgroup (5 cases). Among those with a rebleed, 39% (five patients) had a favorable outcome, while four patients had a poor outcome, and four deaths were reported (29%). No predictive factors were found for the occurrence of a rebleed.

Twenty-one deaths were reported (12%). Of the cases with reported death cause, 53% could be related to ISA rupture (i.e., diffuse infarctions, vasospasms, brain edema). In cases with poor outcome or death, clinical deterioration was due to respiratory complications in 29%, spinal cord infarction in 26%, and vasospasm in 19%. Notably, cases with immediate death, severe comorbidities precluding surgery, and historical cases were grouped with the conservatively managed cases, resulting in an inherent bias to further analysis.

Of note, we found 17 reports with documentation of a thrombosed aneurysm on a pathologic specimen obtained in surgery; possibly corresponding to the spontaneous regression seen on imaging.

Spontaneous ISA regression documented by imaging was associated with good clinical outcome (*p* =.01).

## Discussion

### Incidence, detection and reporting

The true incidence of ISAs is likely underestimated. Case reports from high-volume centers or featuring atypical clinical courses are more likely to be published. Additionally, the current research infrastructure tends to deprioritize or undervalue the publication of case reports. Additionally, rare diseases like ISAs present diagnostic challenges due to a lack of information in medical textbooks, nonspecific clinical symptoms, and subtle imaging findings. Thus, cases with sudden death or those with a mild clinical course with spontaneous symptom resolution may remain undetected. Moreover, spinal imaging is rarely ordered in infratentorial SAH of unclear origin, which could lead to overlooking some ISAs. Estimates of ISA incidence vary, with reports ranging from 1 ISA in 3000 (spinal angiograms for any indication) [6] to 1 ISA in 100 spinal angiograms (performed for SAH cause identification) [7] with our data in the higher range (1:100, 9 ISAs identified in about 500 spinal angiograms for any indication, possibly due to a restricted indication of a spinal angiogram in our centers).

ISAs should be considered in the differential diagnosis of patients presenting with acute, thunderclap-like back pain, focal neurological deficits, headache, nausea, vomiting, or neck stiffness—particularly in the presence of clinical risk factors such as drug abuse, inflammatory or stenotic vasculopathy, connective tissue disorders, prior transplant surgery, chemotherapy, anticoagulation therapy, pregnancy, or prolonged episodes of vomiting.

In cases of cerebral subarachnoid hemorrhage (SAH) of unclear origin — primarily in intraventricular or atypical perimesencephalic distributions — the clinical image and the risk factors should be assessed carefully. In high-risk patients, spinal MRI is warranted. MRI may reveal signs of spinal SAH, spinal cord infarction, or a tubular-cystic enhancing extramedullary structure. Spinal angiography guided by previous CT/MRI findings is indicated in all patients with spinal hemorrhage or suspected spinal vascular lesions. Initial angiographic studies may appear normal, as ISAs can be obscured by vasospasm, intraluminal thrombus, or surrounding hematoma. Follow-up angiography may be necessary to detect aneurysm reperfusion, growth or regression.

Reporting should include precise anatomical information: the level and origin of the parent artery and the downstream artery it supplies. Anatomical descriptions in the literature are often inconsistent. Based on our experience and review of the published imaging, the most common parent vessel is an RMA supplying the ASA, however, many published cases attribute these ISAs to the ASA, likely reflecting variability in anatomical terminology and classification.

High-resolution imaging of small radicular arteries is critical for accurate diagnosis. This can be facilitated by flat-panel detector CT angiography, which provides detailed 3D reconstructions essential for identifying subtle vascular abnormalities.

### Etiology and Pathophysiology

Although a wide range of associated conditions and risk factors have been reported in the literature, it is notable that most patients have an unremarkable medical history or multiple comorbidities without a causal relationship to ISA formation.

Approximately 80% of ISAs are fusiform in morphology, suggesting an underlying pathophysiological mechanism consistent with arterial dissection. This may also account for the relatively high incidence of spontaneous thrombosis observed on follow-up imaging and in histopathological specimens. A potential contributing factor is elevated wall shear stress, which may result from increased blood flow in settings of spinal artery recruitment. Such flow alterations can occur in the context of aortic coarctation [8–11] stenotic vasculopathy [12–14] or following transplant surgery involving artery transposition [15–17]. Other possible mechanisms include vessel wall injury due to inflammatory processes (e.g., vasculitis syndromes [18, 19]sepsis, etc.), or microtrauma from adjacent pathology such as discospondylitis [20, 21] or repeated Valsalva maneuvers associated with prolonged vomiting [22, 23]. In some cases, a combination of these factors may be present, as seen in pregnancy-related ISAs [24, 25]. Rarely, congenital ISAs have been proposed in case reports involving very young children [26, 27]. 

### Treatment approach

The management of isolated spinal aneurysms (ISAs) should be individualized based on the patient’s comorbidities, clinical presentation, and the availability of local expertise. An interdisciplinary approach is essential in tailoring and reevaluating surgical, endovascular, and conservative strategies, or their combination.

Surgical intervention is indicated for spinal cord decompression due to extensive hemorrhage or mass effect of the aneurysm, clinical and imaging deterioration and rebleeding. In cases with extensive spinal cord infarction, treatment decisions should be individualized based on the location of the aneurysm and the assessment of risks and benefits of an intervention. A persisting ISA on follow-up imaging should be considered for surgical or endovascular treatment. In cases with non-treatable risk factors, closer follow-up may be warranted and an interventional approach may be preferred. Surgery may be preferred in posteriorly located aneurysms with easier access and less risk for infarction. In selected cases, temporary occlusion of the parent artery with intraoperative neurophysiological monitoring has been employed to minimize the risk of spinal cord ischemia.

Endovascular approaches include coiling [8, 13] glue [16] and less commonly particle embolization [28]. Endovascular approaches is preferred for anteriorly located ISAs. Endovascular treatment of these fusiform aneurysms is challenging. Parent vessel occlusion is often necessary, with devastating consequences of spinal ischemia. Many ISAs are too small to be treated effectively, while in larger lesions, the limited epidural space may not accommodate coils safely. Moreover, liquid embolic agents carry a substantial risk of spinal cord infarction. Technical limitations such as difficult access to radicular branches and proximal arterial occlusion—as seen in cases of aortic coarctation—can further complicate endovascular approaches. Given the rarity of ISAs, there is a lack of robust data on long-term vessel patency, rebleeding rates, and clinical outcomes following either surgical or endovascular intervention.

### Spontaneous regression

Spontaneous regression of ISAs has been reported more commonly than previously assumed [4, 28–30]. The underlying pathophysiology is likely the thrombosis of a dissecting aneurysm during the process of endothelial healing, possibly facilitated by the slow flow in the small-caliber feeding arteries.

The high rate of favorable clinical outcomes identified supports a trial of conservative management in selected patients with little to no neurological deficits, initial rapid clinical amelioration, multiple ISAs, or a high risk of treatment-related complications due to comorbidities. A thorough clinical work-up is essential to identify treatable risk factors. Regular clinical and imaging follow-ups are critical. If there is a delay between diagnosis and planned intervention, repeat imaging is strongly recommended to assess for spontaneous regression and potentially avoid unnecessary treatment. This approach is different from the established management of ruptured intracranial aneurysms, reflecting the difference in underlying pathophysiology.

**Fig. 3:**
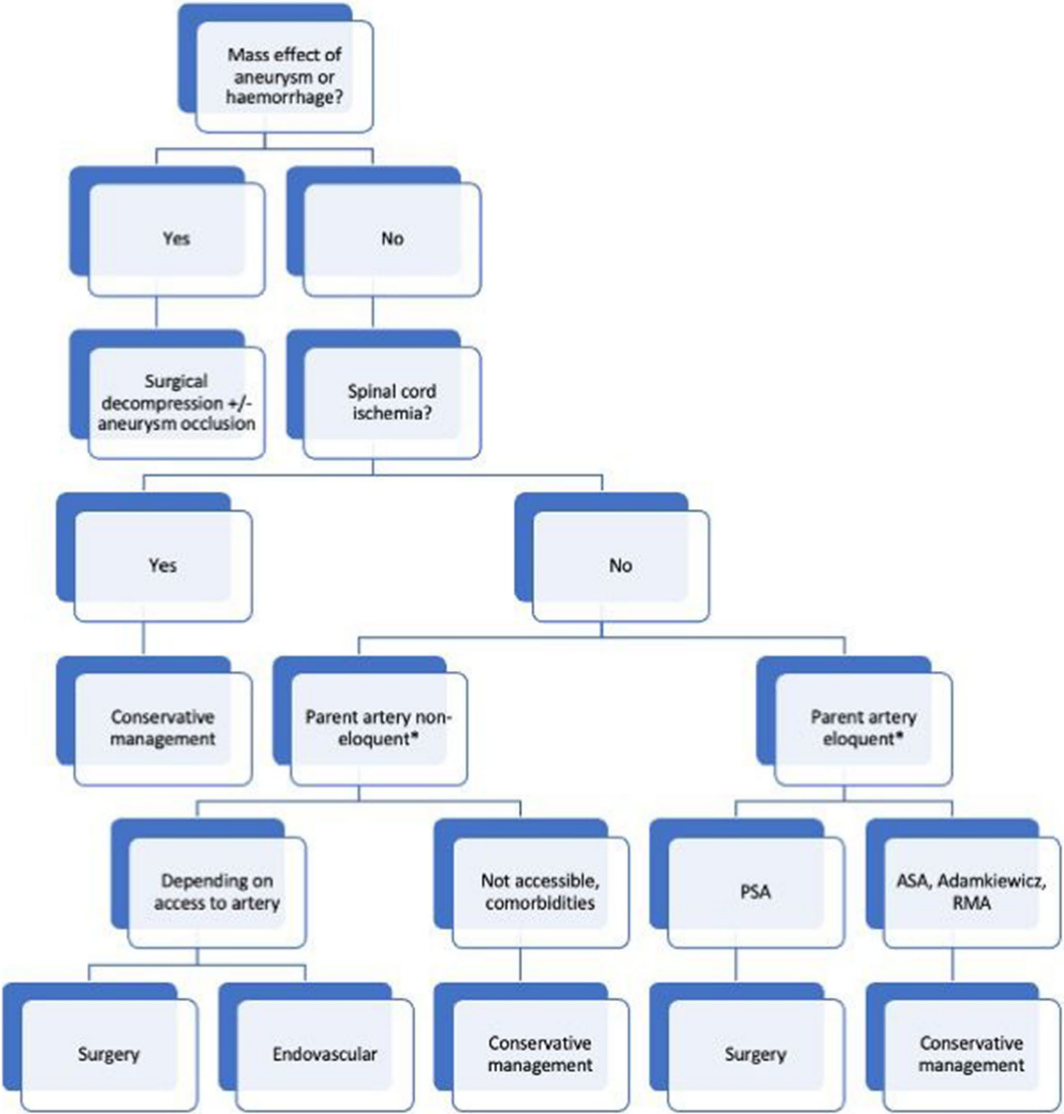
Proposed management algorithm for isolated spinal aneurysms

We conclude these observations in a management algorithm based on our clinical experience and the available evidence (Fig. 3). In cases with neurological deficits due to a mass effect of the hemorrhage or the ISA, surgery is necessary. In cases with early spinal cord infarction, benefits of an interventional approach are very limited. In the absence of mass effect or ischemia, interventional approaches must be weighed depending on the accessibility of the ISA, the type of parent artery and its downflow supply, as well as therapeutic options for comorbidities, and life expectancy. Treatment decisions regarding this rare entity should always be made on a case-by-case basis by a multidisciplinary consensus. If conservative management is opted for, patients should be informed of the necessity of regular clinical and imaging follow-ups.

### Limitations

This work provides an update on the clinical and therapeutic aspects of ISAs. The presented cases were identified through a retrospective review of clinical databases, with an inherent bias of possible underreporting. A major limitation of the case series is the limited follow-up data, as patients were either unable or unwilling to undergo further follow-up examinations. The data from the literature review is inherently subject to publication bias, due to the lack of an organized trial or case database, as reports of atypical or rare observations are more likely to be published, possibly excluding the more common findings. The quality of evidence provided by the literature review is limited by that of the included studies. A meta-analysis was not feasible due to the limited and heterogeneous nature of the available data.

## Conclusions

Isolated spinal aneurysms are a rare and likely underdiagnosed cause of cerebral or spinal subarachnoid hemorrhage, with subtle or underrecognized clinical and imaging features. Their pathophysiologic background and clinical course differ from that of cerebral aneurysms. Conservative management is warranted in selected cases, where spontaneous regression of the aneurysm results in rapid clinical amelioration and an excellent clinical outcome.

## Supplementary Information

Below is the link to the electronic supplementary material.

ESM 1 (DOCX 25.5 KB)

ESM 2 (DOCX 47.1 KB)

## Data Availability

No datasets were generated or analysed during the current study.
