# Supplementary material for: Isolated spinal aneurysms with spontaneous regression
Source: Neurosurg Rev. 2025 Sep 8;48(1):635. doi: 10.1007/s10143-025-03768-8 (PMC12417233; doi:10.1007/s10143-025-03768-8)
Supplement: Supplementary file 1 — (DOCX 25.5 KB) [file 10143_2025_3768_MOESM1_ESM.docx]

| **Reference (PMID if not otherwise specified)** | **Year** | **Age** | **Sex** |
| --- | --- | --- | --- |
| Riforma medica, 15 juillet 1903 an XIX n 28 761 | 1903 | 27 | F |
| Balo J. Über ein Aneurysma der Rückenmarksarterie, welches Tabes-dorsalartige Symptome vortäuschte. Dtsch Z Nervenheilkd 1925;85:86. | 1925 | 63 | M |
| Babonneix M, Widiez A: Sclérose combinee; lésions inflammatoires et diffuses du névraxe. Anévrisme de l'artere spinale anterieure. Syphilis probable. Rev Neurol 1:1214–1217, 1930. | 1930 | 56 | M |
| Echols DH and Holcombe RG Extramedullary aneurysm of the spinal cord New Orleans Medical and Surgical Journal 1941 Vol 93 No 11 p 582 | 1941 | 30 | F |
| Henson R, Croft P: Spontaneous spinal subarachnoid haemorrhage. Q J Med 97:53–66, 1956 | 1956 | 51 | M |
| 13463639 | 1957 | 41 | F |
| 4759663 | 1973 | 40 | M |
| 948020 | 1976 | 25 | F |
| 571987 | 1979 | 34 | F |
| 7420184 | 1980 | 42 | F |
| https://doi.org/10.5694/j.1326-5377.1980.tb134773.x | 1980 | 66 | F |
| 7233470 | 1981 | 30 | F |
| 7099415 | 1982 | 30 | F |
| 6847899 | 1983 | 37 | F |
| 3960303 | 1986 | 29 | M |
| 3810459 | 1987 | 48 | F |
| 3362323 | 1988 | 53 | M |
| 2661726 | 1989 | 17 | F |
| 1432139 | 1992 | 26 | F |
| 1607957 | 1992 | 3 | F |
| 8327110 | 1993 | 40 | M |
| 8355828 | 1993 | 50 | F |
| 8410233 | 1993 | 55 | F |
| 8006673 | 1994 | 59 | F |
| 8971846 | 1997 | 30 | M |
| 10369227 | 1999 | 42 | M |
| 10616066 | 2000 | 28 | F |
| 10952191 | 2000 | 54 | M |
| 11322459 | 2001 | 69 | F |
| 11322459 | 2001 | 72 | F |
| 20594486 | 2002 | 39 | M |
| 15029924 | 2004 | 71 | F |
| 15709145 | 2005 | 69 | F |
| 15709145 | 2005 | 62 | F |
| 15709145 | 2005 | 48 | M |
| 15975794 | 2005 | 74 | F |
| 15987562 | 2005 | 54 | M |
| 16219857 | 2005 | 54 | M |
| 16219857 | 2005 | 69 | M |
| 16219857 | 2005 | 30 | M |
| 16219857 | 2005 | 73 | M |
| 16331160 | 2005 | 69 | M |
| 16331160 | 2005 | 73 | M |
| 16331160 | 2005 | 30 | M |
| 16331160 | 2005 | 54 | M |
| 16619685 | 2006 | 59 | F |
| 16908584 | 2006 | 58 | M |
| 16954077 | 2006 | 56 | F |
| 17176024 | 2006 | 55 | M |
| 35782203 | 2006 | 49 | F |
| 17446995 | 2007 | 30 | F |
| 18074693 | 2007 | 65 | F |
| 18518680 | 2008 | 54 | F |
| 18681926 | 2009 | 55 | F |
| 19209383 | 2009 | 61 | M |
| 19297164 | 2009 | 67 | F |
| 19404125 | 2009 | 46 | F |
| 19558292 | 2009 | 69 | M |
| 19693431 | 2010 | 27 | F |
| 20642894 | 2010 | 43 | M |
| 20726754 | 2010 | 38 | F |
| 20739828 | 2010 | 36 | M |
| 21123989 | 2010 | 51 | M |
| 21990656 | 2010 |  | M |
| local | 2010 | 51 | F |
| 21946730 | 2011 | 60 | F |
| 22257516 | 2011 | 78 | M |
| 22592330 | 2012 | 52 | M |
| 22728865 | 2012 | 72 | F |
| 22869687 | 2012 | 84 | M |
| 22881040 | 2012 | 67 | F |
| 23086108 | 2012 | 67 | F |
| 23197965 | 2012 | 47 | M |
| 23233375 | 2013 | 58 | M |
| 23593602 | 2013 | 47 | M |
| 23903351 | 2013 | 70 | F |
| 23964047 | 2013 | 1 | F |
| 24044082 | 2013 | 45 | F |
| 24436861 | 2013 | 41 | M |
| 22885167 | 2014 | 59 | F |
| 24951379 | 2014 | 30 | F |
| 25317354 | 2014 | 43 | F |
| 25496690 | 2014 | 72 | F |
| 25496690 | 2014 | 37 | F |
| 25006043 | 2015 | 15 | M |
| 25228118 | 2015 | 68 | M |
| 25228118 | 2015 | 51 | F |
| 25809436 | 2015 | 22 | M |
| 25904568 | 2015 | 74 | M |
| 26079782 | 2015 | 72 | M |
| 26154150 | 2015 | 70 | F |
| 26522607 | 2015 | 84 | M |
| 28663963 | 2015 | 59 | F |
| 26230543 | 2016 | 72 | F |
| 26275629 | 2016 | 21 | M |
| 26370306 | 2016 | 55 | F |
| 26582490 | 2016 | 54 | M |
| 26862449 | 2016 | 88 | F |
| 26927604 | 2016 | 38 | M |
| 27114966 | 2016 | 53 | M |
| 27259283 | 2016 | 59 | M |
| 28424013 | 2017 | 46 | M |
| 28424013 | 2017 | 25 | F |
| 28424013 | 2017 | 18 | M |
| 28785998 | 2017 | 9 | M |
| 28966818 | 2017 | 53 | F |
| 29159157 | 2017 | 49 | M |
| 29382773 | 2017 | 45 | F |
| 29382773 | 2017 |  |  |
| 29382773 | 2017 |  |  |
| 29382773 | 2017 |  |  |
| 29382773 | 2017 | 69 | M |
| 29382773 | 2017 |  |  |
| 29382773 | 2017 |  |  |
| 29382773 | 2017 |  |  |
| 29382773 | 2017 |  |  |
| 29382773 | 2017 | 60 | F |
| 29382773 | 2017 |  |  |
| local | 2017 | 41 | F |
| 28987836 | 2018 | 57 | F |
| 28987836 | 2018 | 27 | F |
| 29229349 | 2018 | 78 | M |
| 29627796 | 2018 | 54 | F |
| 30688204 | 2019 | 42 | M |
| 30930328 | 2019 | 54 | F |
| 31029009 | 2019 | 52 | F |
| 31786383 | 2020 | 67 | F |
| 32339730 | 2020 | 36 | M |
| 33505290 | 2020 | 45 | M |
| 32949802 | 2021 | 64 | M |
| 33223127 | 2021 | 75 | M |
| 33223127 | 2021 | 55 | M |
| 33223127 | 2021 | 45 | F |
| 33223127 | 2021 | 65 | F |
| 33535981 | 2021 | 49 |  |
| 33561554 | 2021 | 56 | F |
| 33561554 | 2021 | 37 | F |
| 33561554 | 2021 | 51 | F |
| 33561554 | 2021 | 72 | F |
| 33781941 | 2021 | 63 | F |
| 33932775 | 2021 | 43 | F |
| 34020612 | 2021 | 35 | F |
| 34391976 | 2021 | 77 | F |
| 34482430 | 2021 | 71 | F |
| 35079484 | 2021 | 83 | M |
| 35855466 | 2021 | 76 | F |
| 37502805 | 2021 | 78 | M |
| local | 2021 | 34 | M |
| local | 2021 | 38 | F |
| 33540447 | 2022 | 62 | M |
| 34637940 | 2022 | 35 | F |
| 34637940 | 2022 | 37 | F |
| 35624905 | 2022 | 68 | F |
| 36007976 | 2022 | 65 | M |
| 36281476 | 2022 | 54 | M |
| 36701669 | 2022 | 51 | F |
| local | 2022 | 40 | M |
| local | 2022 | 46 | M |
| 36227207 | 2023 | 54 | M |
| 36628492 | 2023 | 23 | M |
| 36628492 | 2023 | 54 | M |
| 36628492 | 2023 | 72 | F |
| 36628492 | 2023 | 64 | M |
| 36628492 | 2023 | 60 | F |
| 38146935 | 2023 | 48 | M |
| 38335520 | 2024 | 34 | F |
| 38335520 | 2024 | 16 | M |
| 38335520 | 2024 | 57 | M |
| 38335520 | 2024 | 74 | F |
| 38335520 | 2024 | 32 | F |
| 38335520 | 2024 | 57 | M |
| 38335520 | 2024 | 29 | M |
| local | 2024 | 84 | F |
