# Supplementary material for: Isolated spinal aneurysms with spontaneous regression
Source: Neurosurg Rev. 2025 Sep 8;48(1):635. doi: 10.1007/s10143-025-03768-8 (PMC12417233; doi:10.1007/s10143-025-03768-8)
Supplement: Supplementary file 2 — (DOCX 47.1 KB) [file 10143_2025_3768_MOESM2_ESM.docx]

**Identification of studies via databases and registers**

Records removed *before screening*:

Duplicate records removed (n = 46)

Records marked as ineligible by automation tools (n = )

Records removed for other reasons (n = )

Records identified from*:

Databases (n = 436)

**Identification**

Records screened

(n = 390 )

Records excluded**

(n = 42)

Reports sought for retrieval

(n = 348)

Reports not retrieved

(n = 9)

**Screening**

Reports excluded:

Not ISA (n = 226)

Reports assessed for eligibility

(n = 339)

Studies included in review

(n = 127)

**Included**

Flow chart of the process of the systematic literature review according to PRISMA guidelines.

Page MJ, McKenzie JE, Bossuyt PM, Boutron I, Hoffmann TC, Mulrow CD, et al. The PRISMA 2020 statement: an updated guideline for reporting systematic reviews. BMJ 2021;372:n71. doi: 10.1136/bmj.n71
